# Preventing Attacks on Wireless Networks Using SDN Controlled OODA Loops and Cyber Kill Chains

**DOI:** 10.3390/s22239481

**Published:** 2022-12-04

**Authors:** Paul Zanna, Peter Radcliffe, Dinesh Kumar

**Affiliations:** School of Electrical and Electronic Engineering, RMIT University, Melbourne, VIC 3000, Australia

**Keywords:** IEEE 802.11, wifi, denial-of-service, security, software defined networking, P4

## Abstract

Impersonation-based attacks on wireless networks are easy to perform and can significantly impact network security. Their detection is problematic due to the attacks utilizing legitimate functions. This paper proposes a novel algorithm based on Observe-Orientate-Decide-Act (OODA) loop and Cyber Kill Chain (CKC) strategies to detect and neutralize these attacks. To evaluate this approach, we conducted experiments using four attack methods on a wireless router equivalent device, five wireless client devices, and two attack devices. The system employs a Radio Frequency (RF) device identification system and attack state machine implemented using a Software Defined Networking (SDN) architecture and the P4 programming language. The technique remains compliant with the IEEE 802.11 standard and requires no client-side modifications. The results show that the RF section detected 97.5% (average) of impersonated frames, and the overall method neutralized all attacks in the four attack scenarios. This outcome demonstrates that this technique, built on the OODA loops and CKC methodology, using SDN architecture and P4, is suitable for real-time detection and prevention of wireless impersonation attacks.

## 1. Introduction

Defending a wireless network from impersonation attacks is difficult as they commonly exploit features inherent to the ongoing management [1] of an IEEE 802.11 network session. The management frames used to establish or terminate client connections and authentication handshakes are prime targets for attackers. The susceptibility of management frames to attack is due to the fact that they are unauthenticated and unencrypted in all versions prior to WPA3. This inherent lack of management frame security enables attackers to masquerade as legitimate clients, disrupting connectivity.

The IEEE 802.11w amendment in 2009 [2] introduced the concept of Protected Management Frames (PMF) by defining a group of Robust Management Frames. This group comprises various post-authentication frames, such as de-authentication, disassociation, and control frames, which are the most vulnerable to attack. This amendment, which provides authentication of management frames, protects against all described attacks. However, its implementation is complex due to the lack of support amongst current generation devices. Moreover, PMF has been shown to have vulnerabilities [3]. Unfortunately, the latest version of the IEEE 802.11 standard WPA3, which mandates PMF, could not resolve the issue due to backward compatibility with the previous weaker versions [4]. Additionally, it has been found that WPA3 is still susceptible to various attacks [5]. With methods that attempt to counteract attacks on a standards-based service, the difficulty is creating a practical defense solution that remains compliant with the endorsed standards.

Attacks specific to wireless networks are focused on the PHY and MAC layers of the network stack and usually fail into one of three types. First, a Denial-of-Service (DOS) attack, which can target either OSI layer, is designed to overwhelm a particular client or an entire network. Second, attacks targeting credentials or encryption keys can also use techniques from other attack types as a precursor. Finally, those designed for eavesdropping on user traffic may overlap with credential attacks. While the methods of attack on wireless networks vary, examples of which are shown in Table 1, many share the same underlying mechanisms [1]. Furthermore, this table references the previously proposed solutions, many of which are too complex and resource-intensive for a WAP or require modifications to the IEEE 802.11 standard.

The ability to defend against impersonation attacks relies on two capabilities, detection and prevention [17]. First, the device must determine the difference between legitimate and nefarious commands. Second, it should be able to absorb, deflect or neutralize these attacks. While incorporating both features together would provide a holistic solution, these features are often deployed separately based on the examined literature. Detection functionality can be provided independently by standalone sensors or integrated directly into the wireless infrastructure. In comparison, defensive functionality must be implemented at the point of frame processing.

Detecting impersonation attacks within a wireless network is complex; therefore, the advantage is with the attacker due to the simplicity of launching these attacks [18]. The difficulty in detecting and preventing impersonation attacks originates from the fact that many are based on the abuse of authorized commands within the IEEE 802.11 standard. Moreover, these standards are binary and do not differentiate between a command issued once or hundreds of times in rapid succession. Therefore, this situation presents the challenge of continuing to offer the services within the applicable standards while being able to determine what appropriate and inappropriate behaviors are. 

**Figure 1:**
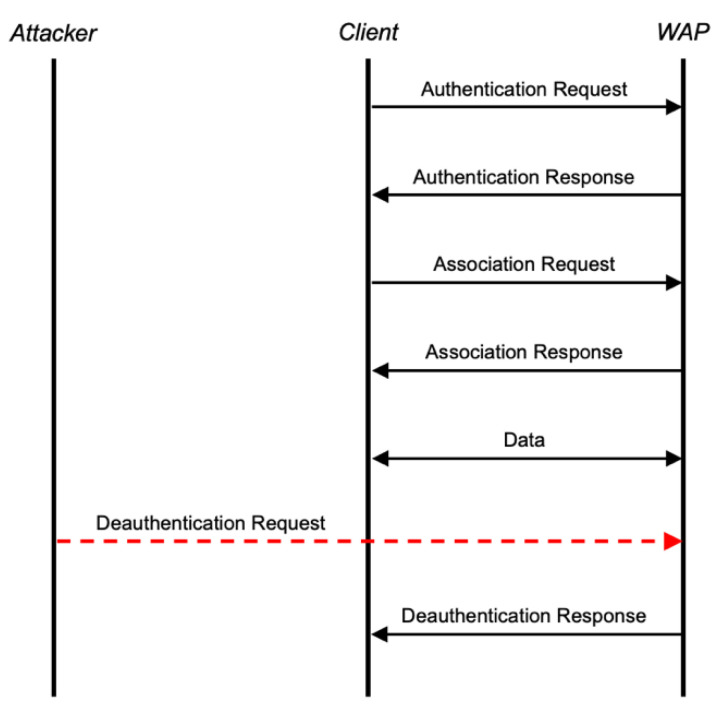
Deauthentication attack process.

The deauthentication attack is one of the most common and effortlessly executed attacks [1]. This attack is designed to disconnect a client from a Wireless Access Point (WAP) and, if continued repeatedly, can render a network unusable for one or all client devices. The interactions between a client, WAP, and attacker, as shown in Figure 1, outline the process used by which an attacker can disrupt connectivity between the client and WAP by falsely signaling to the WAP that the client wishes to end the session. A deauthentication request is a standard command sent from a client to a WAP or vice versa to advise the other party that the session is no longer required. From an attack vector, it can be utilized in two ways. It can disconnect clients, forcing them to reauthenticate, allowing an attacker to capture the authentication handshake for offline cracking. Alternatively, it can be used as a pure DOS attack against one or more clients connected to a WAP by repeatedly issuing the command each time a client reconnects [10].

One concept commonly used by cybersecurity professionals is the Cyber Kill Chain (CKC) [19,20,21]. This concept was later extended by Wilkens et al. [22], who demonstrated the approach using a Kill Chain State Machine (KCSM). A CKC defines the order of tasks used by a third party to execute an attack. Additionally, the concept of Situation Awareness (SA), defined as understanding current threat and attack status, was described by Barford et al. [23] as having at least seven aspects:

- be aware of the situation;
- be aware of the impact of an attack;
- be aware of how situations evolve;
- be aware of actor (adversary) behavior;
- be aware of why and how the current situation is caused;
- be aware of the quality (trustworthiness of information);
- assess plausible future states.

Barford et al. later consolidated these seven levels into three phases: Perception, Comprehension, and Projection. These authors describe what they refer to as the “dream” system that can determine its situational awareness and protect itself when under attack without human intervention. However, they state that this system is far from possible with current technologies. Moreover, Andrade demonstrated the same three phases as Yoo [24] to align with another military model, the Observe-Orient-Decide-Act (OODA) loop developed by the United States Air Force Colonel John Boyd [25]. The tenant behind the OODA loop is completing a loop faster than the opponent, preventing them from gaining superiority in combat.

In the same way, if a defender can act more quickly than an attacker in a cybersecurity conflict, they can achieve “cyber superiority” [26]. As Dykstra and Orr describe in comparison to their Cynefin model [27], “the OODA loop encourages agility and speed to react to our opponents.” This makes it possible to create a tactical advantage by discovering intent and the individual steps in their CKC. 

As Table 1 details, the literature provides thirteen methods for addressing IEEE 802.11 attacks, yet the problem remains. This persistent gap is primarily due to Radio Frequency (RF) fingerprinting options requiring high-end hardware to support resource-intensive algorithms [28]. Therefore, in this paper, we describe a complete method to overcome the current limitations of the inability of small resourced wireless devices to defend against impersonation attacks. This approach incorporates the OODA and CKC methodologies as a situational state machine and the Multiplexed One-Class Classifier identification algorithm as a novel method called the Wireless Impersonation Detection and Defense (WIDD) to detect and actively defend against a range of IEEE 802.11 attacks. Based on the OODA loop and CKC, WIDD uses a systematic approach to detect, evaluate, and respond to threats while being transparent to the user and sufficiently lightweight to run on a commodity access point.

The novelty of the approach described in this paper is:

- integration of the Multiplexed One-Class Classifier (MOCC), which previously demonstrated a high degree of accuracy, into a P4 application as a device identification algorithm;
- the use of the Software Defined Networking (SDN) programming language P4 [29] to deliver a novel method for detecting and defending a WAP from impersonation attacks;
- a novel algorithm that defends against simultaneous attacks of different types per-client basis demonstrated in a real-world setting using commonly used attack tools such as Aircrack-ng [30] and Fluxion [31].

We will discuss the related work and compare our contributions in Section 4.

## 2. Solution Overview

**Figure 2:**
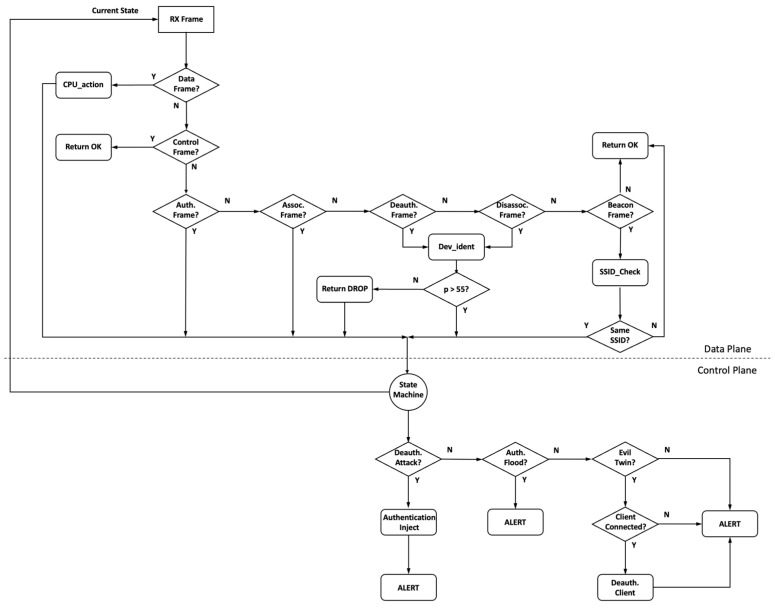
P4 flow diagram.

The WIDD architecture consists of three key components: A Kill Chain State Machine, P4 helper APIs, and active countermeasures. Each component is controlled by an OODA loop algorithm, shown in Figure 2, created using the P4 programming language. It is this algorithm that determines the appropriate Kill Chain logic to apply. The IEEE 802.11 frames are forwarded from a customized version of the Openwifi FPGA implementation which has been modified to capture the RF features used by the MOCC. The frame and RF feature set are sent to the P4 packet parser stage, which determines the frame type. Control frames are returned to the wireless driver, whereas data frames, which are authenticated and thus not from an attacker, are directed to the MOCC using the P4 *CPU_action* call. The MOCC uses these data frames to create a set of classifier rules to build a device signature. Management frames which are the basis for most IEEE 802.11 attacks are processed differently.

Management frames used for authentication and association can form part of a flood-type attack designed to overwhelm the WAP and impact network performance. Therefore, when either of these frames is received, the algorithm updates the flood attack KCSM. Moreover, the WAP drivers have been modified to capture the beacon frames transmitted by other WAPs, which are then sent to the *SSID_check* API to determine whether any other WAPs are broadcasting the same SSID. Rogue access points broadcast false SSID beacons to entice legitimate clients to connect, capturing their login credentials or providing network connectivity that can be used for eavesdropping. Again, the KCSM is updated to provide real-time responses. Finally, deauthentication frames, used in one of the most common DOS attacks, are checked against the classifier model of known clients using the *Dev_ident* API. Those frames that return probability below the determined threshold (initially *p* < 55%) are dropped, whereas those above are processed normally with the KCSM updated to track multi-frame or multi-client attacks.

Based on the KCSM output, and a set of Kill Chain triggers, the P4 logic delivers appropriate countermeasures. For deauthentication attacks, false authentication frames are transmitted as attackers commonly force re-authentication using deauthentication frames to capture the login handshake. Authentication and association flood attacks trigger an alert in addition to dropping the illegitimate frames. Finally, where evil twin or rogue access points have been detected, a warning is generated, and periodic deauthentication frames are transmitted using the legitimate client and rogue WAP’s MAC addresses to reduce the likelihood of eavesdropping. 

### 2.1. OODA Loop

**Figure 3:**
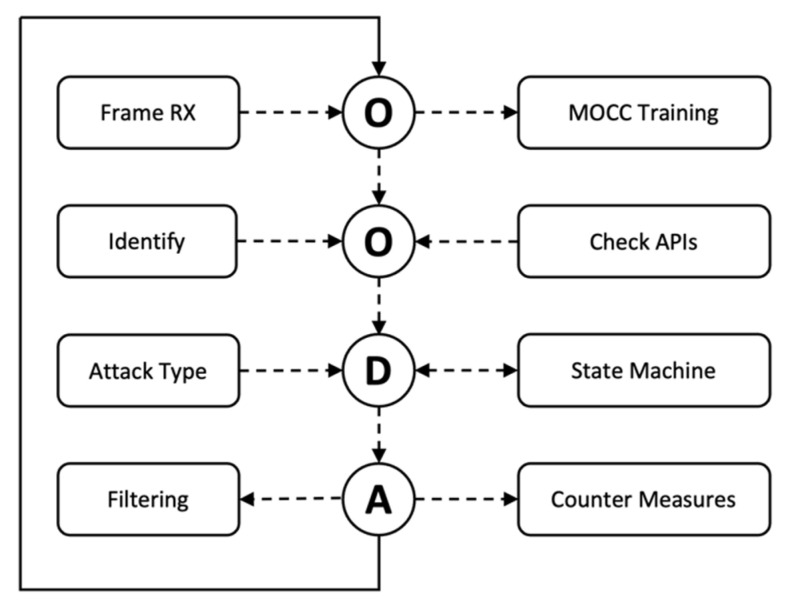
OODA loop functions.

The OODA loop implementation in Figure 3 is a series of steps implemented in P4 conditional logic shown in Appendix A, a subset of the overall source code [32]. 

Each stage is executed sequentially upon receiving a frame from the Openwifi SDR driver. The “Observe” stage receives the frame via the P4 interface and, using the header fields, determines the frame type: Data, Control or Management.

Data frames are sent to the MOCC training algorithm via the *CPU_action* API. Control frames are not utilized and therefore returned to the SDR driver for processing in the usual manner. Management frames, the primary means of initiating attacks, are sent to the “Orientate” stage.

The Orientate stage determines the management frame type and decides which support API function to enlist. The output of these support functions is then combined with frame-type logic to produce an input to the “Decide” stage. The decide stage is a set of state machines that maintain inter-frame states, allowing the WAP to determine the correct type of action. The state machine accepts the input shown in Table 2, which is produced as an outcome of the orientate stage of the OODA loop logic. These state machine inputs are also shown in Figure 2 as outputs of the data plane logic.

The final stage of the OODA loop is the action stage, where the possible countermeasure options are executed. The action taken is determined by the output of the state machine, as shown in Table 3. Countermeasures may include dropping frames, forwarding frames or injecting new frames to neutralize attacks. Once the action stage is complete, the cycle restarts upon receiving a new frame.

### 2.2. P4 Support API Functions

Data frame headers which can only be from an authenticated use, use the *CPU_action* API to ensure the MOCC algorithm’s successful training. The four RF characteristics, RSSI, Phase Offset, Pilot Offset, and Magnitude Squared, are included with the header to build a fingerprint of each known device. These headers are sent to the control plane via a FIFO frame buffer for use by the learning algorithm. It was previously found that 600–1000 frames are required to produce a fingerprint with an identification accuracy greater than 98%. The time to reach this frame count varies significantly based on the device’s type and usage profile. For example, computers and smartphones streaming video could achieve this volume in minutes, whereas an IoT device that only sends periodic updates could take days.

The *Dev_ident* function is the prediction component of the MOCC and is used to calculate a device identity probability based on the classifier rules created by the learning algorithm. Again, from the previous testing, it was found that *p* > 55% certainty is the optimal cutoff point based on sensitivity and specificity and therefore used to determine the identity flag in the decision stage of the OODA loop.

The *SSID_check* API is then used to determine the SSID contained within received beacon frames. While beacon frames from other WAPs are typically ignored, the FPGA and driver were modified to allow these frames to be received and processed by the P4 code. As P4 contains no string evaluation functionality, this feature requires an external API-based capability. The SSID is extracted from the beacon frame’s Information Element (IE) and then compared to the SSID of the WAP. If the SSID values match, yet the BSSID is different, another WAP broadcasts the same wireless network name and may be part of the Evil Twin or MITM attack. 

The *State_update* API provides the observe and orientate logic and returns an action. The function also receives the client’s MAC address to enable separate states to be maintained per device. A state update message is loaded into the frame buffer used by the *CPU_action* API to transfer frame headers to the control plane, thereby sharing a single integration point between data and control planes.

### 2.3. Kill Chain State Machine

**Figure 4:**
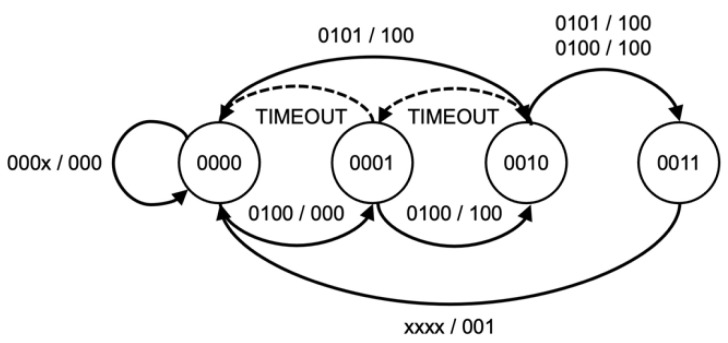
Deauthentication KCSM.

**Figure 5:**
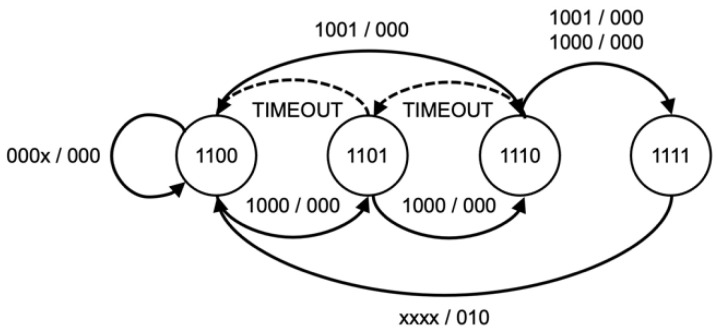
Disassociation KCSM.

**Figure 6:**
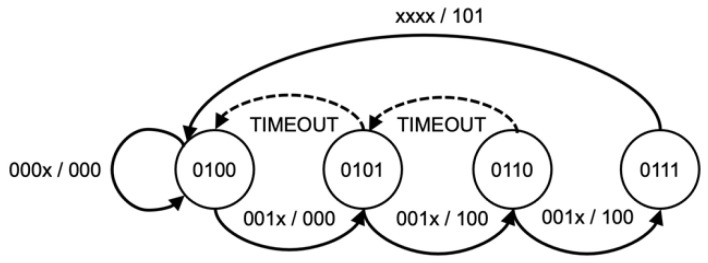
Authentication flood KCSM.

**Figure 7:**
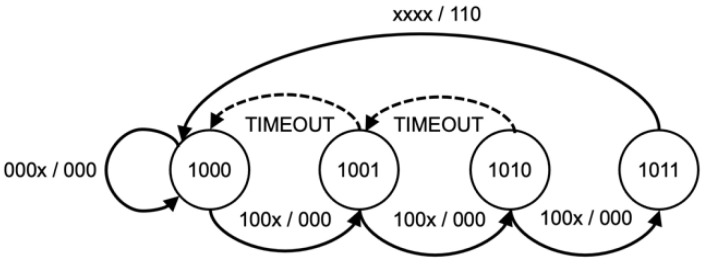
Association flood KCSM.

The KCSM, a series of parallel state machines, were implemented based on predetermined attack techniques used to maintain the current phase of a Kill Chain, as shown in Figure 4, Figure 5, Figure 6 and Figure 7. While Wilkens et al. applied the model at a broad network level, this approach refines and extends the concept in a novel way to the individual frame level.

The sequence and status of each attack kill chain are stored in a set of state machines accessed via a P4 support API function. The state of each attack is stored on a per-client basis allowing for the processing of concurrent attack phases. This method enables the WAP to track and counteract multiple attacks of different types or even attacks that may simultaneously target some or all client devices. This algorithm synchronizes each wireless client’s state to detect coordinated attacks intelligently. The memory footprint and processing requirements are minimal, utilizing only 512 bytes of state memory. Each state sequence is based upon a kill chain model of the attack sequence and customized to allow for timing between states and variations due to wireless anomalies, such as dropped or corrupt frames. As each kill chain is different, the state model will be explained in further detail in the evaluation section below.

### 2.4. Countermeasures

The actions are initiated based on the State Machine output shown in Table 3. These actions protect WAP availability and credential security or prevent clients from falling victim to MITM attacks. While nearly all the solutions described in Section 4 employ various passive measures for minimizing attacks, we propose two novel active countermeasures that defend against attacks and disable the attacker’s ability to perform these attacks. These are disconnecting the known clients connected to a rogue WAP broadcasting false SSIDs and sending false authentication handshake messages after deauthentication attacks to disrupt credential cracking kill chains.

## 3. Evaluation

Evaluating the proposed solution in a way that provides a representative use case requires using WAP equivalent hardware and real-world attack methods. Therefore, to demonstrate the effectiveness of the WIDD algorithm, we performed a series of attacks against the WAP test hardware, running our modified version of Openwifi [33]. As this is a complete end-to-end solution, it must use real-time over-the-air test data to allow the RF features to be evaluated by the MOCC identification algorithm. Therefore, it is not possible to use preexisting or standardized datasets for evaluation.

The test hardware is the Analog Devices ADRV9361-Z7035 [34] which includes all the required functionality and suitable capacity requirements of the Openwifi software. This device consists of a Xilinx Zynq 7035-2L SoC Dual-core ARM Cortex-A9 running at 800 MHz, making it the hardware equivalent of a mid-range domestic wireless router. The instance of Openwifi used had been previously extended to include MOCC, which uses a specially developed rule-based classifier, a P4 application interface, and a customized Orthogonal Frequency Division Multiplex (OFDM) library to facilitate the use of various RF features for identification and behavioral analysis.

Five devices were on the client side of the testbed, including laptops, smartphones, and IoT devices. Each device was connected to the WAP on 5 Ghz, on channel 44, using 802.11a, although the 2.4 Ghz band functionality is equivalent. After authentication, a minimum of 5000 data frames were collected to create a usable ruleset for the classifier. Attacks were generated from a single laptop using two different USB WIFI dongles with chipsets from Realtek and Broadcom (TP-Link WDN3200), thereby providing a variety of RF signatures for comparison. The attack device was running Kali Linux, and the Aircrack suite of tools was used to create the DOS attacks. This approach is the easiest and most common method of attack and therefore the same method used by real-world attackers. Furthermore, Fluxion was used as an alternate method of performing Evil Twin and MITM attacks.

### 3.1. Deauthentication/Disassociation DOS Attacks

Deauthentication attacks are relatively simple to perform using a tool such as Aireply-ng, which produces various customizable management frames. The command *“aireplay-ng -0 1 -c FF:EE:DD:CC:BB:AA -a 66:55:44:33:22:11 wlan0”* was used to transmit a group of 64 deauthentication frames, instructing the evaluation WAP to deauthenticate the client with the specified MAC address. Five commonly used wireless clients and an Acer laptop with two wireless dongles as a simulator attacker were used in this study, as shown in Table 4. The success rate of detecting false deauthentication frames shows a high accuracy rate apart from the MacBook Air and Realtek combination. For this reason, detection is a two-part process; identification is only the first part of the detection algorithm.

The second part of the Deauthentication and Disassociation attack detection process is provided by the KCSM, as shown in Figure 4 and Figure 5. The attack state is updated based on the number of valid or false deauthentication frames. Under normal circumstances, a client will transmit a single deauthentication frame when disconnecting from a WAP. Therefore, the receipt of more than one frame could be considered to constitute an attack. However, to reduce the risk of false negatives, two false deauthentication frames or three total deauthentication frames received within 2 s are required to complete the state transitions and trigger an attack state. The 2-s-window was chosen as it is the deauthentication timeout value [35] set by *hostapd*, a commonly used control and authentication daemon on Linux based WAPs. This approach also compensates for any poor device identification results, an example of which can be seen in Table 4 with the Realtek and MacBook Air combination. While it is possible for a client to be erroneously disconnected due to an incorrectly identified deauthentication frame, the continued receipt of these frames will trigger an attack state allowing the client to reconnect and stay connected, thereby mitigating a DOS outcome.

### 3.2. Credential Attacks

The next attack type, Credential Cracking, requires performing a password dictionary attack. However, the attacker must first capture the four-way authentication handshake to compare the key to a rainbow table or password list. This handshake can be captured during standard client authentication; however, this may take some time, thus most attackers first force a reauthentication using a deauthentication attack. Airodump-ng is commonly used to capture the handshake before passing it to Aircrack-ng for cracking. The WIDD uses a novel approach that transmits a false four-way handshake whenever an impersonated deauthentication request is received. This causes the attacker to capture an invalid handshake without knowing the difference, concluding the attack on the belief that they have the necessary key information. Additionally, the handshake frames are transmitted twice to improve reliability and allow for the possibility of the first frame not being received.

To evaluate the effectiveness of this concept, an attack was performed on a client connected to the evaluation WAP. Using Airodump-ng and Aireplay-ng in the same manner as an attacker, a deauthentication attack was performed, and transmission of the four-way handshake was monitored. The handshake was passed to Aircrack-ng and compared to a password list containing only the correct WAP password to validate that the invalid handshake was indeed the one the attacker received. Aircrack-ng failed to determine the password in all tests where the countermeasure was enabled; however, when the countermeasure was disabled, the password was always decrypted correctly.

### 3.3. Evil Twin/Rogue Access Point

The first part of our testing for an Evil Twin or Rogue Access Point attack was the detection of false beacons. As previously described, the frame filtering has been modified to receive beacons from other WAPs, a capability usually only available when a wireless adaptor is in monitor mode. The first test used Airbase-ng to simulate a rogue AP which broadcasts the legitimate SSID with the BSSID, which is the MAC address of the attacker’s wireless network interface. The evaluation platform detected the false SSID/BSSID combination during all tests.

The second part of our test used the Fluxion tool, an automated MITM attack platform. Fluxion uses a series of steps to scan for SSIDs, capture authentication handshakes, and launch a captive portal complete with a web server and DNS redirect. Fluxion does not perform a brute-force crack of the handshake key; instead, it tricks the user into thinking they are being prompted for their credentials using the captive portal. When the user enters their password, it is compared to the captured handshake key, and if it matches, it is logged. The user is then disconnected and redirected back to their legitimate WAP without realizing they have just given away their password.

When Fluxion was started, it scanned for SSIDs, returning a list that included the SSID of the evaluation platform. Once the evaluation SSID was selected and Fluxion began broadcasting the false beacon, it was detected, and an alert was raised. While this would usually be the end of this attack, the warning was ignored in order that the attack could continue allowing for later stages of the WIDD to be tested. The next step is to capture a four-way handshake. Fluxion uses the same approach as the credential crack above, sending deauthentication frames to disconnect the client and logging the handshake when the client reconnects. Accordingly, the WIDD detected the false deauthentication frame and transmitted the invalid handshake frames causing Fluxion to log incorrect credentials. Therefore, even when false SSID detection was disabled and the client connected to Fluxion by entering the correct password, it did not match, stopping Fluxion from validating the password. Additionally, Fluxion was executed with the two different wireless adaptor types, both of which produced the same outcome, a complete attack failure. Finally, Fluxion was run against the evaluation platform with the WIDD features disabled to ensure an effective test setup, and it could successfully comprise a user’s credentials.

### 3.4. Authentication/Association Flood Attacks

An authentication flood is, again, a simple yet effective DOS attack that requires little knowledge on behalf of the attacker. This was performed using the Aireply-ng tool with the authentication frame command, *“aireplay-ng -1 0 -e openwifi -h FF:EE:DD:CC:BB:AA -a 66:55:44:33:22:11 wlan0”,* which transmits a continuous stream of false authentication frames. While a relatively simple attack to detect was underway, genuine authentication requests can be challenging to differentiate from malicious ones. Flood attacks were performed using all client and attacker device combinations, shown in Table 4, and were also successfully detected and mitigated.

## 4. Discussion

The novel approach presented herein produces the required outcome by incorporating RF device identification, an attack progress state machine derived from kill chain and defense methodologies, and active countermeasures, all controlled by a unique application of the P4 programming language. Each of these components expands upon and differentiates itself from the literature in multiple ways. Therefore, this section will not only compare these mechanisms but also discuss the unique way in which these features have coalesced.

The device identification technique described in this paper incorporates all four RF features used by the MOCC, thereby reducing its susceptibility to deliberate signal strength modulation. Xu et al. [36] have shown that RSSI values can vary considerably with movement and environmental changes. One option to overcome this is a multi-node, MAC layer spoofing approach, such as the one offered by Sheng et al. [37]. While using a Gaussian Mixture Model from over twenty sensors proposed by Sheng et al. may improve detectability in large environments, it is not viable for small deployments. However, as stated earlier, not relying on RSSI alone is a far superior approach to device differentiation.

Using a state machine may resemble counting deauthentication requests, similar to the one demonstrated by Baharudin et al. [38]. However, their approach only detected rudimentary brute force style attacks and did not carefully craft credential capture attacks, which are more likely to resemble normal behavior [1]. For this reason, including the novel countermeasures we propose, such as transmitting a false four-way handshake, demonstrates a better defense outcome than simply counting deauthentication requests.

The SDN paradigm as a DOS attack detection solution previously proposed by Cwalinski and Koenig [39] uses a method based on their “RADIator” framework [40]. This system employs the Channel State Information (CSI) to help identify clients based on proximity, in a similar way to [41,42]. However, location-based solutions are subject to various issues, such as small-scale fading due to movement [36]. While the work proposes a DOS attack detection solution, it contains very little information on using a location-based fingerprinting solution for detecting DOS attacks. Similarly, Nagarajan et al. [43] examine a method for varying the client transmission power to known levels to identify MAC address spoofing. However, we believe it suffers the same shortcomings as other RSSI methods, namely movement and environmental impacts.

The method of Manjunath et al., which proposed in their patent [44] the prevention of intruder attacks, is based on tricking an attacker into sending deauthentication requests from a MAC address known to be false, which triggers an alert when receiving a deauthentication request from one of these random addresses. However, an attacker needs only verify a MAC address using MAC addresses from data frames that are part of a two-way exchange, not single management frames, thereby ensuring they are part of a valid, authenticated session. Within the same patent, the authors also outline RSSI values to correlate the sender of a deauthentication request with the actual client. However, many deauthentication attackers vary the signal strength in order that deauthentication requests do not appear to come from the same device.

The target under attack needs not always be the WAP, as deauthentication attacks can also be directed at a wireless client. The patent lodged by Sundaram et al. [45] describes a method for detecting deauthentication attacks on a client by monitoring deauthentication requests sent to a client and reconciling those with commands issued directly by the WAP. Additionally, the patent describes identifying attacking devices using fingerprinting and then using this fingerprint to block all traffic. Moreover, the solution alerts administrators of the device’s possible location based on the signal strength and triangulation information from multiple WAPs. Unfortunately, as with most patents, the authors provide no information on the effectiveness of this approach or its useability in a real-world environment. Furthermore, it is very similar to an IDS, which requires additional hardware and ongoing support, which is unnecessary for the operation of WIDD.

Tamhane et al. [46] described that an active validation method employs the Fine Time Measurement (FTM) function to confirm a deauthentication request that a client has issued. The FTM feature was introduced in the 2016 update of the IEEE 802.11 standard and has since become a common method for range evaluations [47,48,49]. The authors state that it is possible to validate the command upon receiving a deauthentication request by sending an FTM request from a wireless controller to the client. If two or more responses are received, another device impersonates the client. The wireless controller determines the actual client based on the round-trip response time intervals upon receiving more than a single reply. The model has some merit, although it relies on a newer standard not supported on many low-end devices. Additionally, it leaves many scenarios unaccounted for, such as purpose-built deauthentication devices that do not respond to the false MAC addresses they impersonate. Furthermore, the possible impact of message floods and the resources required to validate each may cause exhaustion, further exacerbating the problem.

Detecting other DOS attacks follows a similar methodology; the goal is to differentiate the real devices from the impersonations. For example, an individual device taking on multiple identities is achieved by a Sybil attack. While these attacks are more common in ad-hoc networks, the literature on detection is similar to many other wireless DOS attacks. Consequently, Wang et al. [50] employed the method to detect Sybil attacks by adding CSI to improve previous RSSI-only methods to determine the forged devices based on location and movement. While the authors’ detection algorithm showed an accuracy of over 98% under ideal conditions, the authors reported flaws in the method when people without wireless devices are moving around within the environment. Again, the use of RSSI or CSI has been repeatedly shown to be flawed; even the human body has an impact on radio propagation where it has been shown to influence CSI values, to a great extent that it has been utilized as an environmental tracking method [51].

DOS attack detection methods that utilize machine learning at a protocol layer, such as Doshi et al. [52], demonstrate the use of lightweight anomaly detection algorithms in IoT networks. These authors evaluated five machine learning models to determine when a DOS attack was underway. At the same time, variation in performance and accuracy across the different models highlighted the significant effort required to separate an attack from regular traffic. In contrast, wireless network attacks are more predictable and, therefore, easier to codify using state machines, as demonstrated here. Similarly, Li et al. [53] used various TCP/IP features of IoT devices and five different machine learning algorithms, using the same Scikit-learn Python library [54] as Doshi et al. Similarly, Alipour et al. [55] used IEEE 802.11 MAC-layer behavior analysis to implement a Wireless Intrusion Detection System (WIDS) that monitored device authentication state transitions. Unfortunately, the disadvantage of this and many other DOS prevention techniques is their passivity; most operate in a standalone monitor fashion or, if deployed in line with traffic processing, use a pass if GOOD, drop if BAD methodology, leaving them at the attacker’s mercy. For this reason, we decided to take a more dynamic approach to prevent and neutralize wireless attacks using active countermeasures.

Initial approaches to defending against deauthentication attacks, such as the approach suggested by Bellardo and Savage [10], proposed delaying the processing of deauthentication requests by 10–15 s. While this may reduce the impact of deauthentication attacks, it could also introduce a new set of problems. They described how waiting 10–15 seconds for additional frames to be sent by the client, which, if received, would signal they had no intention of ending the session and indicate the possibility of a third party. Although this approach is simple and may reduce some fraudulent requests’ impact, it introduces a new issue with session handoff (roaming) when multiple access points are in use. Additionally, the overhead of maintaining a state for numerous clients could be a new point of exhaustion attack.

Martínez et al. [14] suggested that one of the most efficient techniques to detect beacon spoofing attacks is to develop profiles of the wireless devices to create behavior-based anomaly detection. Nevertheless, they also argue that this method can generate a large number of false positives, asserting this is due to the unstable nature of the wireless medium and the difficulty in modeling the behavior of a diverse range of devices. Their approach claims to combine several techniques to reduce false positives by identifying the impersonation of specific management frames. However, the method they describe simply uses an external sensor to measure the intervals between beacon frames, referred to as the Delta, and uses this to detect spoofed beacons. Unfortunately, the solution requires additional hardware, and the paper does not describe the effect of lost frames, the accuracy at various distances or signal strengths. Conversely, our proposed solution can readily determine externally transmitted beacon frames as it is part of the WAP’s beacon transmission pipeline.

As beacon frames are transmitted every 102.4 ms according to the IEEE 802.11 standard, Amoordon et al. [56] suggested that a shorter interval may signify the presence of multiple access points broadcasting the same SSID. The approach, which the authors demonstrated as effective in limited tests, also required an auxiliary device to act as an Intrusion Detection System (IDS). The approach of using an external device was due to the fact that they believed it was not a function the WAP could execute, a misconception our work has proven. Amoordon et al. then extended their IDS method [57] by evaluating seven ML algorithms to determine whether the RSSI values of the beacon frames could provide additional accuracy. They argued that no single approach could detect rogue access points, jamming, and deauthentication attacks simultaneously and that their IDS could perform this, which is an assertion this work also disproves.

While a fundamental consideration of the solution detailed herein was to remain compliant with IEEE 802.11 standard, this is not always the case with other approaches previously proposed. For example, Ananay Arora [58] and Noman et al. [12] proposed adding a unique ID to deauthentication frames to verify authenticity. Similarly, Nguyen et al. [59] developed a letter-envelope protocol to add signing capabilities to the deauthentication frames. The Malekzadeh et al. [60] proposal includes a keyed message authentication code. As these proposed solutions require a deviation from the IEEE 802.11 standard and a client-side modification, they become incompatible with off-the-shelf devices and therefore restricted in their usage. While these approaches may appear relatively simplistic, they raise the question of using bespoke methods rather than the previously accepted PMF standard. Additionally, the difference in processing the overhead of these methods compared to PMF is an outstanding question.

## 5. Conclusions

Attackers can easily compromise IEEE 802.11 wireless networks using readily available tools and minimal skills. While the literature provides diverse approaches to addressing these attacks, many are complex and require processing beyond the capabilities of most WAPs and home routers. This overhead considerably restricts the usability and, in turn, the breadth of deployment options. Given that the attacks described herein could impact all IEEE 802.11 devices, large and small, the goal is to support the lowest possible requirements while still being compliant with IEEE 802.11 standards, which would increase the protection footprint significantly.

We have presented an impersonation attack detection and defense methodology incorporating a behavioral algorithm derived from the OODA loop and CKC strategies. This model is combined with an RF device identification function and integrated into an access point with domestic grade specifications. This approach creates an automated, zero-touch solution to protect a wireless network against the most common impersonation attacks. The method overcomes the previous techniques’ high-end or external equipment requirements. Additionally, the approach requires no client modification and remains within the IEEE 802.11 standard. This conformity to universally agreed standards allows the demonstrated solution to protect even the most rudimentary clients, such as IoT devices, without changing their compatibility. Finally, using the P4 programming language allows for further enhancement, which is directly compatible with other P4 devices, supporting endless extensibility and reusability. The results show the effectiveness of this technique in detecting and preventing these attacks in real-time.

## Figures and Tables

**Table 1:** Examples of IEEE 802.11 attacks.

Wireless Attack	Type	Vector	Layer	Target
Frequency Offset [6]	DOS	Impersonation	PHY	WAP
Preamble SYNC [7]	DOS	Impersonation	PHY	WAP
RTS/CTS [8,9]	DOS	Flow Control	MAC	Ad-hoc
Power Saving Mode [10,11,12]	DOS	Power Management	MAC	WAP/Client
IEEE 802.11w Deadlock [13]	DOS	Authentication	MAC	WAP
Deauthentication [10]	Impersonation/DOS	Authentication	MAC	WAP/Client
Disassociation [10]	Impersonation/DOS	Association	MAC	WAP/Client
Beacon Flood [14]	DOS	Impersonation	MAC	Client
Authentication/Association Flood [12]	DOS	Impersonation	MAC	WAP
Sybil [15]	DOS	Impersonation	MAC	WAP/Client
Evil Twin/Rogue Access Point [16]	Impersonation	MITM	MAC	Client
Cafe Latte [1]	Credential	ARP	MAC	Client
Dragon Blood [4]	Credential	Side Channel	MAC	WAP/Client

**Table 2:** State machine inputs.

Inputs	*Q* *_a_*	*Q_b_*	*Q_c_*	*Q_d_*
Data Frame	0	0	0	x
Authentication	0	0	1	x
Deauthentication (False)	0	1	0	1
Deauthentication (True)	0	1	0	0
Evil Twin Beacon	0	1	1	x
Association	1	0	0	x
Disassociation (False)	1	0	1	1
Disassociation (True)	1	0	1	0

**Table 3:** State machine outputs.

Outputs	*K_a_*	*K_b_*	*K_c_*
No Attack	0	0	0
Deauthentication Attack	0	0	1
Disassociation Attack	0	1	0
Evil Twin	0	1	1
Credential Attack	1	0	0
Authentication Flood	1	0	1
Association Flood	1	1	0

**Table 4:** RF device identification accuracy percentage.

	Attacker	TP-Link WDN3200	Generic Realtek 8812BU
Client			
iPhone 12 (2021)		100%	84.4%
MacBook Pro (2013)		100%	100%
HP TPN-C126 Laptop (2017)		100%	98.4%
MacBook Air (2020)		100%	7.8%
Samsung Galaxy 10e (2019)		87.5%	95.3%
Average		97.5%	77.2%

## Data Availability

Not applicable.

## Appendix A

OODA loop algorithm as P4 source code.
state_input = state_read(); // Management frame if (headers.frameCtrl.frameType == 0x0) { if (headers.frameCtrl.subType == 0xB) state_output = state_update(AUTH); if (headers.frameCtrl.subType == 0x0) state_output = state_update(ASSOC); // De-authentication Frame if (headers.frameCtrl.subType == 0xC) { lookup_tbl.apply(); // Deauthentication frame from a known device. if (ID_ok == 1) state_output = state_update(DEAUTH + VALID_ID); // Deauthentication frame from an unknown device. if (ID_ok == 0) { state_output = state_update(DEAUTH); Drop_action(); } } // Disassociation Frame if (headers.frameCtrl.subType == 0xA) { lookup_tbl.apply(); // Disassociation frame from a known device. if (ID_ok == 1) state_output = state_update(DISASSOC + VALID_ID); // Disassociation frame from an unknown device. if (ID_ok == 0) { state_output = state_update(DISASSOC); Drop_action(); } } // Beacon Frame if (headers.frameCtrl.subType == 0x8) { ssid = ssid_check(headers); // Beacon frame with the same SSID but different BSSID, Evil Twin!! if (ssid == 1) state_output = state_update(BEACON); // Beacon frame with different SSID, another WAP in range - ignore. Pass_action(); } } if (headers.frameCtrl.frameType == 0x1) // Control frame. { Pass_action(); // Pass the frame. } if (headers.frameCtrl.frameType == 0x2 && state_input == 0x0) // Data frame. { CPU_action(); state_output = state_update(DATA); }
